# Light-mediated temperature susceptibility of kelp species (*Agarum clathratum*, *Saccharina latissima*) in an Arctic summer heatwave scenario

**DOI:** 10.1017/cft.2024.5

**Published:** 2024-03-13

**Authors:** Sarina Niedzwiedz, Tobias Reiner Vonnahme, Thomas Juul-Pedersen, Kai Bischof, Nora Diehl

**Affiliations:** 1 Marine Botany, Faculty of Biology and Chemistry & MARUM, University of Bremen, Bremen, Germany; 2 Greenland Climate Research Centre, Greenland Institute for Natural Resources (GINR), Nuuk, Greenland

**Keywords:** climate change, coastal ecology, marine ecosystems, sedimentation, water temperature

## Abstract

Kelps (Phaeophyceae, Laminariales) are ecosystem engineers along Arctic rocky shores. With ongoing climate change, the frequency and intensity of marine heatwaves are increasing. Further, extensive meltwater plumes darken Arctic fjords. Assessing the effect of a sudden temperature increase at the cold-distribution limit of cold-temperate kelp species, we compared the responses of two kelp species (*Agarum clathratum*, *Saccharina latissima*) to realistic Arctic summer heatwave scenarios (4–10°C) under low- and high-light conditions (3; 120 μmol photons m^−2^ s^−1^) for 12 days. We found high-light causing physiological stress in both species (e.g., lower photosynthetic efficiency of photosystem II), which was enhanced by cold and mitigated by warm temperatures. Under low-light conditions, we found no temperature response, probably due to light limitation. Both species acclimated to light variations by adjusting their chlorophyll *a* concentration, meeting cellular energy requirements. *A. clathratum* had ~150% higher phlorotannin concentrations than *S. latissima*, possibly acting as herbivore-deterrent. Our findings suggest competitive advantages of kelps on different Arctic coasts with ongoing warming: *A. clathratum* has advantages in future areas, with low-light intensities, and possibly high grazing pressure and *S. latissima* in areas with high-light intensities and low grazing pressure. Species composition changes might have cascading consequences on ecosystem functioning.

## Graphical abstract

**Figure:**
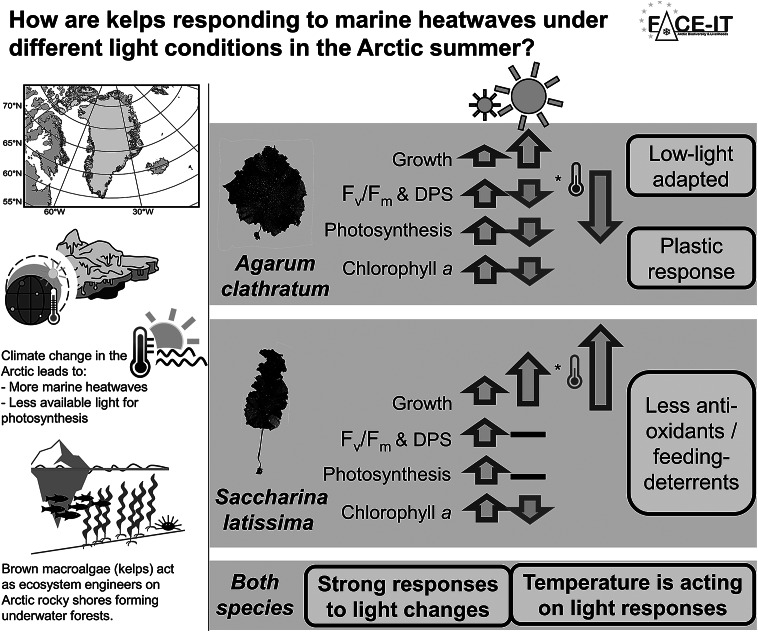

## Impact statement

Kelps are brown macroalgae that act as ecosystem engineers on many rocky shore coastlines from temperate to polar regions, covering about 25% of the global coastline. They provide habitat, food, and nursery ground for many associated species, some of which are economically relevant. Kelps in the Arctic experience various climate change related environmental variations, such as intense, sudden short-term temperature increases (heatwave), or a reduction of the available light for photosynthesis due to glacial meltwater with high sediment concentrations being washed into fjords. To be able to preserve these valuable and vulnerable ecosystems, it is important to understand how species dynamics change, responding to single and interacting drivers. In this study, we worked in Nuup Kangerlua, Greenland, assessing how a heatwave affects the sieve kelp (*Agarum clathratum*) and sugar kelp (*Saccharina latissima*), two cold-temperate kelp species, when being exposed to either high-light (clear Arctic fjord) or low-light (meltwater covered Arctic fjord) conditions. We found high-light conditions to inflict most physiological stress in both species, being amplified by cold (in situ) temperatures. Warm temperature during the heatwave scenario had mitigating effects. This finding supports existing models on expansion of temperate kelps to higher latitudes with rising temperatures. When kelps were low-light-limited, temperature had no effect on either species response. Low-light intensities resulted in significantly reduced net photosynthetic rates, indicating less overall production and a reduced contribution to the coastal carbon cycle of kelps. Our results suggest that each kelp species will have competitive advantages in different Arctic coastal areas with increasing warming: The sieve kelp has competitive advantages in areas, with low-light intensities, and possibly high grazing pressure and the sugar kelp in areas with high-light intensities and low grazing pressure. This might have cascading consequences on ecosystem functioning, affecting species-dependent associated species or energy transfer to higher trophic levels.

## Introduction

Kelps (Laminariales, Phaeophyceae) form underwater forests on rocky shore coastlines in temperate and polar regions. They act as foundation species and ecosystem engineers, providing nursery ground, habitat, and food for many associated species (Eckman et al., 1989; Filbee-Dexter et al., 2019; Smale, 2019; Wernberg et al., 2019). Covering about 25% of the world’s coastlines, kelp forests provide many socioeconomic services (Teagle et al., 2017; Wernberg et al., 2019). However, being sedentary, kelps cannot actively escape stressors and are susceptible to environmental changes (Ruthrof et al., 2018; Straub et al., 2019). Accordingly, kelp species developed a large range of physiological and biochemical strategies to acclimate to changes in the environment (Hurd et al., 2014). Regarding temperature, for example, each species has a specific physiological optimum within which they exhibit maximal performance at lowest energetic costs (Pörtner et al., 2005). Above or below the optimum, cellular stress and energetic costs are increasing, resulting in decreasing performance (Kültz, 2005).

Temperature is considered a major driver for the latitudinal distribution of kelp species (Lüning, 1990; Adey and Steneck, 2001; Fragkopoulou et al., 2022), as it is directly affecting enzymatic activities (Clarke and Fraser, 2004; Pörtner and Farrell, 2008). Within their genetically set tolerance limits, kelp species can acclimate to increasing temperature by modifying their phenotype (King et al., 2018, Liesner et al., 2020).

As a consequence of climate change, the mean global sea surface temperature is already 0.88°C (0.68–1.01°C) higher comparing 2011–2020 to 1850–1900, with further increasing tendencies (IPCC, 2023). These global changes have consequences for a species entire biogeographical distribution range, as a shift in temperature causes shifts in their performance (e.g., growth) along their reaction norm (Chevin et al., 2010). However, most drastic consequences of rising temperatures become apparent at the species warm and cold distribution limits: Kelp forests were observed to disappear at their warm-edge distribution limits (Sorte et al., 2010; Filbee-Dexter and Wernberg, 2018), as genetically adaptive modifications of the temperature tolerance limits over generations were shown to mismatch with the pace of projected temperature changes (Vranken et al., 2021). With the Arctic warming far beyond the global average (Previdi et al., 2020, 2021; England et al., 2021), habitats in high latitudes become (increasingly more) habitable for temperate kelp species. Both processes combined, a passive northward shift of temperate kelp species is predicted (Assis et al., 2022), potentially outcompeting and replacing cryophilic Arctic kelp species (Bringloe et al., 2020, 2022).

Additional to the long-term temperature increase, the frequency and intensity of extreme temperature events, such as marine heatwaves (MHWs), are expected to increase (Hobday et al., 2016; Oliver et al., 2018; Barkhordarian et al., 2024). Thereby, MHWs are defined as a temperature increase above the 90th percentile of the 30 year mean for more than five consecutive days (Hobday et al., 2016). MHWs have drastic consequences for ecosystems, triggering mortality, demographic and species community disruptions and might be the tipping point for alternative ecosystem states (Wernberg et al., 2016; Filbee-Dexter and Wernberg, 2018; Straub et al., 2019). Filbee-Dexter et al. (2020) highlighted that kelp forest declines in the Atlantic coincided with increasing intensities and frequencies of MHWs, being replaced with low-productive turf algae. Overall, Smale (2019) and Wernberg et al. (2013) evaluated warming and marine heatwaves as major threat for kelp forests worldwide. Further, it was found that even if kelp abundances did not decline due to immediate heat stress, their susceptibility to other stressors increased (Wernberg et al., 2010).

In Arctic coastal areas, elevated temperatures also alter the underwater light availability to primary producers. Increased temperatures lead to an early season breakup of sea ice (increased light availability; Nicolaus et al., 2012; Payne and Roesler, 2019), while higher terrestrial runoff (Bintanja and Andry, 2017; Milner et al., 2017; Bintanja, 2018) decreases light availability in summer (Gattuso et al., 2020; Konik et al., 2021). Thereby, Schlegel et al. (2023) describe a high interannual variability of light availability. As photoautotrophic organisms, kelps are dependent on the underwater light conditions, driving their depth distribution (Fragkopoulou et al., 2022). They can only grow if their carbon uptake exceeds their carbon loss (Kirk, 2011). The interaction of changes of the underwater light conditions and in temperature is especially important for kelp distribution, as photosynthetic processes are driven by a multitude of (temperature-sensitive) enzymatic reactions (Davison et al., 1991). Therefore, increasing temperatures might also affect kelps’ light tolerance.

*Agarum clathratum* (Nova Scotia; Simonson et al., 2015) and *Saccharina latissima* (Helgoland; Bolton and Lüning, 1982; *S. longicruris* morphology: Long Island; Egan et al., 1989) were described to have a temperature optimum between 10 and 15°C. Fortes and Lüning (1980) monitored *S. latissima* to survive periods at 0°C and Bringloe et al. (2022) classified *A. clathratum* as cryotolerant in areas with temperatures below 0°C. Based on these findings, Nuup Kangerlua (SW Greenland) with a mean annual temperature (upper 5 m water column) of 1.97°C (GEM, 2023) is at the northern cold limit of both species. Nonetheless, it is important to note reported optimum temperature ranges can vary for one species, depending on environmental conditions and geographical location (Bennett et al., 2019). Though this is not evident for *A. clathratum* or *S. latissima* yet.

**Figure 1.**
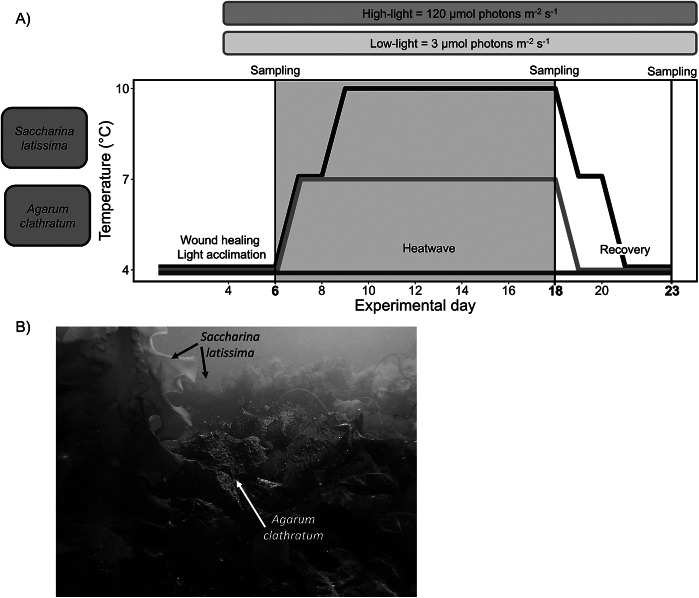
(A) The kelp species *Agarum clathratum* and *Saccharina latissima* were exposed to heatwave scenarios (blue: control, 4°C; orange: 7°C heatwave; red: 10°C heatwave) under low-light (3 μmol photons m^−2^ s^−1^) and high-light (120 μmol photons m^−2^ s^−1^) conditions. Days 0–5: wound healing and light acclimation. Days 6–18 (grey): heatwave. Days 19–23: recovery. The photosynthetic efficiency of photosystem II was measured every 2 days; all other parameters were measured on days 6, 18, and 23. (B) In situ photo of the kelp forest around Nuuk, showing *Agarum clathratum* and *Saccharina latissima.* © Sarina Niedzwiedz.

In this study, we assessed the acclimation of Arctic *A. clathratum* and *S. latissima* in response to summer heatwaves under different light conditions. Thereby, we provide a more detailed understanding of the future dynamics of kelp forests at their northern cold-distribution limit. Based on abiotic in situ measurements, we conducted a 12-day heatwave simulation experiment (4, 7, 10°C) under low light (3 μmol photons m^−2^ s^−1^) and high light (120 μmol photons m^−2^ s^−1^), evaluating physiological (growth, photosynthetic efficiency of photosystem II (F_v_/F_m_), dark respiration rates, net photosynthetic rates), and biochemical (pigments, phlorotannins) parameters. The heatwave was followed by a 5-day recovery phase (Figure 1A). Our study was guided by three hypotheses:

1. *A. clathratum* was observed to grow deeper in the water column (Figure 1B; video transect see Supplement S1). Hence, we expect it to be adapted to low-light conditions, becoming evident by maintaining high physiological performance at low-light conditions.
2. Given the described temperature optimum of 10–15°C (Bolton and Lüning, 1982; Egan et al., 1989; Simonson et al., 2015), we expect an increase in performance of both species with rising temperature.
3. Suboptimal temperatures will increase cellular energy demand (Pörtner et al., 2005). Given the reported temperature tolerance ranges, we consider cold, in situ temperatures suboptimal. Low-light conditions might not provide enough energy. Hence, we hypothesize that the interaction of cold temperatures and low-light causes physiological stress.

## Material and methods

### Study region, sampling, and experimental setup

Nuup Kangerlua is located between N 64 and 65° south-western Greenland. Similar sized sporophytes of *Agarum clathratum* (Dumortier, 1822) were sampled at low tide between 8 and 10 m and *Saccharina latissima* (Lane et al., 2006) between 7 and 8 m at N 64.203° W 51.648° (tidal range: >2 m; Richter et al., 2011). We collected the “*longicruris*” morphology of *S. latissima* (= hollow, very long stipe), contrary to the classic “*latissima*” morphology (= solid, short stipe). McDevit and Saunders (2010) detected no genetic differentiation between both morphotypes. Egan et al. (1989) found a temperature optimum for growth at 10–15°C for “*S. longicruris*” (consistently with Bolton and Lüning (1982) for *S. latissima*). Hence, we consider them the same species – *S. latissima.* We were granted sampling permission by the Government of Greenland under the nonexclusive license no. G23–007.

Meristematic disks (diameter 2 cm; ~10 per individual) were equally distributed between interacting light and temperature treatments (*n* = 4), avoiding pseudoreplication. The disks were cultivated at 24 h LED light in 2 L aerated plastic beakers, filled with fresh, unfiltered seawater (changed every second day, S_A_ 35). All interacting light-temperature treatments were started in parallel after a 3-day wound healing of experimental specimens and 2 days of light acclimation at 4°C (in situ temperature). Within the heatwave phase (days 6–18), temperatures gradually increased until treatment temperatures were reached. On day 18, a heatwave recovery was conducted with a gradual temperature decrease until reaching 4°C (Figure 1).

Treatment conditions were based on the 15 years’ time series of the Greenland-Ecosystem-Monitoring database (GEM, 2023). Mean summer temperature (June–August) in Nuup Kangerlua in the upper 15 m was 4.2°C. The maximum recorded temperature was 8.5°C in July 2012. Temperature exceeding 7°C were found in July and August in 7 years during the 15 years of monitoring (Supplement S2). Hence, we chose temperature treatments of 4°C (control), 7°C (upper present summer temperature), and 10°C (future temperature). Photosynthetically available radiation (PAR) is typically highest in July with light intensities between ~160 and 2,500 μmol photons m^−2^ s^−1^ at 1 m and ~ 15 to 190 μmol photons m^−2^ s^−1^ at 15 m (GEM, 2023). Referring to the sampling depth, we chose 24 h of 120 μmol photons m^−2^ s^−1^ as high-light conditions (Supplement S2). The chosen low-light conditions (3 μmol photons m^−2^ s^−1^) should represent light conditions within meltwater plumes on 15 m depth. We used the average light attenuation in sediment-plume-dominated Kongsfjorden, Svalbard as reference (Niedzwiedz and Bischof, 2023a,b; Supplement S3).

### Response parameters

All kelp response parameters were measured before (day 6), at the peak of (day 18) and after (day 23) the heatwave (Figure 1). Additionally, maximum quantum yield of photosystem II (F_v_/F_m_) measurements were measured every second day in *n* = 4. F_v_/F_m_ is a proxy for algal physiological and cellular stress (Murchie and Lawson, 2013) and was measured using pulse amplitude modulated fluorometry (Portable Chlorophyll Fluorometer PAM-2100, Heinz Walz GmbH, Effeltrich, Germany) after 5 min of darkness.

Algal growth (dry weight (g) of freeze-dried disks) is an integrative parameter reflecting an organism’s response to the interaction of all environmental parameters.

Dark respiration rates are a proxy for cellular energy requirements. Net photosynthetic rates indicate chemical energy availability of organisms. Both were measured as oxygen concentration evolution responding to different light intensities with a 4-channel optode setup (FireStingO_2_ Fibre-Optic Oxygen Meter FSO2-C4, PyroScience Sensor technology, Aachen, Germany). As incubation chambers 25-mL Schott bottles were used. The system was one-point-calibrated according to the manufacturers’ protocol. A magnetic stirrer ensured homogenic oxygen concentrations. Raw data were corrected for temperature and atmospheric pressure variations using the PyroScience-Calculation-Tool. Linear functions were fitted on the oxygen concentration evolution to calculate the dark respiration and net photosynthetic rates. Linearity of slopes indicated that incubations were not substrate-limited. As we used unfiltered sea water in our experiment, the temperature-dependent mean background (microbes and phytoplankton) respiration and photosynthesis (*n* = 4 per treatment condition) were subtracted. Dark respiration and net photosynthetic rates were normalized to wet weight (g; WW).

Algal pigment content reacts to energy requirements and light availability (Blain and Shears, 2019). Pigment analysis was conducted according to Koch et al. (2015). Thirty to one hundred milligram powdered, freeze-dry material was extracted in 1 mL 90% acetone (v/v) for 24 h in darkness at 4°C. The supernatant was filtered and analyzed by a high-performance liquid chromatography (HPLC; LaChromElite® system, L-2200 autosampler (chilled), DA-detector L-2450; VWR-Hitachi International GmbH, Darmstadt, Germany). A gradient was applied according to Wright et al. (1991), separating the pigments by a Spherisorb® ODS-2 column (250 × 4.6 mm, 5 μm; Waters, Milford, MA, USA). The respective standard for each pigment (DHI Lab Products, Hørsholm, Denmark) was used to identify and quantify pigment peaks. Accessory pigments (Acc) were defined as the sum of chlorophyll *c*, fucoxanthin, and ß-carotin, and the ratio to chlorophyll *a* was calculated (Acc:Chl*a*). The ratio of the xanthophyll cycle pigments, namely the de-epoxidation state (DPS), was calculated after Colombo-Pallotta et al. (2006).

Phlorotannins serve as a mechanism of protection against abiotic stressors and herbivores (Amsler et al., 2009). Total phlorotannin content was determined with the Folin–Ciocalteu assay (Cruces et al., 2013). Twelve to fifteen milligram powdered, freeze-dry material was extracted in 70% acetone (v/v) for 24 h in darkness at 4°C and constant shaking. Two hundred and fifty microliter dH_2_O, 200 μL 20% sodium carbonate (Na_2_CO_3_), and 100 μL 2-N-Folin–Ciocalteu reagent (Sigma-Aldrich, Germany) were added to the extract, incubating 45 min in darkness before measuring the absorbance at 730 nm in a microplate reader (FLUOstar OPTIMA, BMG Labtech). The total phlorotannin content was calculated by using a phloroglucinol dilution series (C_6_H_6_O_3_, Sigma-Aldrich: 0–1,000 μg mL^−1^).

### Statistics

Statistical analyses were run in RStudio (Version 2023.06.0; R Core Team, 2021). Each treatment consisted of *n* = 4 biological independent replicates. Normality (Shapiro–Wilk test, *p* > 0.05) and homoscedasticity (Levene’s test, *p* > 0.05) of the raw data and model’s residuals were tested. As requirements were met, a linear model was fitted on the data, using the “lm” function (Package “stats”; R Core Team, 2021). Temperature (4; 7; 10°C), light (3; 120 μmol photons m^−2^ s^−1^), species (*A. clathratum; S. latissima*), and day (6; 18; 23) were modelled as multiple fixed effects. The model’s fit on the data was assessed. Analysis of variance (ANOVA) was tested on the model by using the “anova” function, assessing the influence of fixed effects. All *p*-values were fdr-corrected for multiple testing, using “p.adjust” (Package: “stats”; R Core Team, 2021). Pairwise comparisons were performed, using the “emmeans” function (Package: emmeans; Lenth, 2021). The level of significance was set to *p* < 0.05. Using Pearson correlation, linear dependency between response variables was tested (function: cor.test; R Core Team, 2021), after testing for normality.

## Results

For overview reasons, all statistical results are shown in Table 1 (ANOVA) and Table 2 (pairwise comparisons) and therefore not given in the text or plots. The fixed effects are abbreviated as follows: temperature – T; light – L; species – S; day – D. All values in the text are gives as mean ± standard deviation.

**Table 1.** Statistical results for all physiological and biochemical parameters. Results of the analysis of variance (ANOVA) to evaluate the effects of the fixed parameter temperature (T), light (L), species (S), and day (D), as well as their interactions on physiological and biochemical parameters. Significant results are highlighted in bold (*p* < 0.05)

Parameter	Fixed effects	numDF	denDF	*F* value	*p* value
Dry weight	**T**	2	108	5.93	**0.009**
	**L**	1	108	155.24	**0.003**
	**S**	1	108	29.33	**0.003**
	**D**	2	108	229.88	**0.003**
	T × L	2	108	8.0	**0.003**
	T × S × D	4	108	0.68	0.76
	L × S × D	2	108	0.1	0.92
	T × L × D	4	108	1.93	0.18
	T × L × S × D	4	108	0.27	0.92
Maximum quantum yield of photosystem II (F_v_/F_m_)	**T**	2	108	33.55	**0.003**
	**L**	1	108	295.44	**0.003**
	**S**	1	108	92.53	**0.003**
	**D**	2	108	5.36	**0.013**
	T × L	2	108	22.9	**0.003**
	T × S × D	4	108	1.44	0.3
	**L × S × D**	2	108	6.1	**0.007**
	T × L × D	4	108	1.54	0.32
	T × L × S × D	4	108	0.59	0.78
Dark respiration rate	**T**	2	108	12.76	**0.003**
	**L**	1	108	59.4	**0.003**
	**S**	1	108	52.2	**0.003**
	**D**	2	108	34.5	**0.003**
	T × L	2	108	0.65	0.68
	**T × S × D**	4	108	3.96	**0.01**
	L × S × D	2	108	0.75	0.64
	T × L × D	4	108	1.12	0.51
	T × L × S × D	4	108	2.74	0.05
Net photosynthetic rate	**T**	2	108	9.93	**0.003**
	**L**	1	108	408.96	**0.003**
	**S**	1	108	27.81	**0.003**
	D	2	108	2.61	0.15
	T × L	2	108	2.5	0.16
	T × S × D	4	108	0.52	0.79
	**L × S × D**	2	108	9.79	**0.002**
	T × L × D	4	108	0.80	0.68
	T × L × S × D	4	108	0.33	0.91
Chlorophyll *a*	T	2	108	2.40	0.2
	**L**	1	108	115.44	**0.003**
	**S**	1	108	118.50	**0.003**
	D	2	108	2.35	0.2
	T × L	2	108	1.56	0.35
	T × S × D	4	108	0.59	0.78
	L × S × D	2	108	2.79	0.13
	T × L × D	4	108	0.67	0.66
	T × L × S × D	4	108	1.11	0.52
De–epoxidation state (DPS)	**T**	2	108	6.9	**0.005**
	**L**	1	108	345.64	**0.003**
	**S**	1	108	167.16	**0.003**
	**D**	2	108	40.1	**0.003**
	T × L	2	108	5.27	**0.013**
	T × S × D	4	108	0.6	0.79
	**L × S × D**	2	108	15.4	**0.003**
	T × L × D	4	108	0.92	0.64
	T × L × S × D	4	108	0.40	0.87
Phlorotannins	**T**	2	108	4.45	**0.02**
	L	1	108	0.95	0.50
	**S**	1	108	148.17	**0.003**
	**D**	2	108	4.68	**0.02**
	T × L	2	108	0.14	0.91
	T × S × D	4	108	0.53	0.79
	L × S × D	2	108	0.06	0.95
	T × L × D	4	108	0.58	0.78
	T × L × S × D	4	108	0.61	0.78

*Note*: Tested values are the mean of means of replicates (*n* = 4). numDF: numerator degrees of freedom. denDF: denominator degrees of freedom.

**Table 2.** Summary of the impact of the interactive fixed effects (T: temperature; L: light; S: species; D: day) on pairwise comparisons of the physiology and biochemistry of kelps (*n* = 4). DW: growth as dry weight. F_v_/F_m_: maximum quantum yield of photosystem II. Resp: dark respiration rate. PS: net photosynthetic rate. Chl*a*: chlorophyll *a.* DPS: de-epoxidation state of xanthophyll cycle pigments. Phl: total phlorotannin content

T	L	S	D	DW	F_v_/F_m_	Resp	PS	Chl*a*	DPS	Phl.
TEMPERATURE	Low	Acla	6			–	–	–	–	–
	Low	Acla	18	–	–	(4 = 7) < 10	–	–	–	–
	Low	Acla	23	–	4 > 10	(4 = 7) > 10	–	–	–	–
	High	Acla	6	–	4 < (7 = 10)	(4 = 7) > 10	4 < 10	–	4 > (7 = 10)	–
	High	Acla	18	–	4 < (7 = 10)	–	4 < 10	–	4 > 7	–
	High	Acla	23	–	(4 = 10) < 7	7 > 10	–	–	–	–
	Low	Slat	6	–	–	(4 = 7) > 10	–	–	–	–
	Low	Slat	18		–	–	–	–	–	–
	Low	Slat	23	–	–	4 > 10	–	–	–	–
	High	Slat	6	–	4 < 7	–	–	–	–	–
	High	Slat	18	4 < 10	4 < (7 = 10)	–	–	(4 = 7) < 10	–	–
	High	Slat	23	4 < 7 < 10	4 < (7 = 10)	7 > 10	–	–	–	–
4	LIGHT	Acla	6	–	L > H	L < H	L < H	–	L < H	–
4		Acla	18	–	L > H	L < H	L < H	L > H	L < H	–
4		Acla	23	L < H	L > H	–	L < H	L > H	L < H	–
7		Acla	6	–	–	L < H	L < H	–	–	–
7		Acla	18	L < H	L > H	L < H	L < H	L > H	L < H	–
7		Acla	23	L < H	L > H	–	L < H	L > H	L < H	–
10		Acla	6	–	–	–	L < H	–	–	–
10		Acla	18	L < H	L > H	–	L < H	L > H	L < H	–
10		Acla	23	L < H	L > H	L < H	L < H	L > H	L < H	–
4		Slat	6	–	L > H	–	L < H	–	L < H	–
4		Slat	18	L < H	L > H	L < H	L < H	L > H	L < H	–
4		Slat	23	L < H	L > H	–	L < H	L > H	L < H	–
7		Slat	6	–	–	–	L < H	–	–	–
7		Slat	18	L < H	L > H	–	L < H	L > H	–	–
7		Slat	23	L < H	L > H	L < H	L < H	–	L < H	–
10		Slat	6	–	L > H	L < H	L < H	–	–	–
10		Slat	18	L < H	–	L < H	L < H	–	–	–
10		Slat	23	L < H	L > H	L < H	L < H	L > H	L < H	–
4	Low	SPECIES	6	–	Acla < Slat	–	–	Acla > Slat		Acla > Slat
4	Low		18	–	–	–	–	Acla > Slat		Acla > Slat
4	Low		23	–	–	–	–	Acla > Slat		Acla > Slat
4	High		6	–	Acla < Slat	Acla > Slat	Acla > Slat	Acla > Slat	Acla > Slat	Acla > Slat
4	High		18	–	Acla < Slat	–	–	Acla > Slat	Acla > Slat	Acla > Slat
4	High		23	–	Acla < Slat	–	–	–	Acla > Slat	Acla > Slat
7	Low		6	–	–	–	–	Acla > Slat		–
7	Low		18	–	–	Acla > Slat	–	Acla > Slat		–
7	Low		23	–	–	Acla < Slat	–	Acla > Slat		Acla > Slat
7	High		6	–	–	–	Acla > Slat	–		Acla > Slat
7	High		18	–	Acla < Slat	Acla > Slat	–	–	Acla > Slat	Acla > Slat
7	High		23	–	Acla < Slat	–	–	–	Acla > Slat	Acla > Slat
10	Low		6	–	Acla < Slat	Acla > Slat	–	Acla > Slat		–
10	Low		18	–	–	Acla > Slat	–	Acla > Slat		Acla > Slat
10	Low		23	Acla < Slat	Acla < Slat	–	–	Acla > Slat		Acla > Slat
10	High		6	–	–	–	Acla > Slat	Acla > Slat		–
10	High		18	Acla < Slat	Acla < Slat	Acla > Slat	–	–	Acla > Slat	Acla > Slat
10	High		23	Acla < Slat	Acla < Slat	–	–	–	Acla > Slat	Acla > Slat
4	Low	Acla	DAY	6 < (18 = 23)	6 < (18 = 23)	–		–		–
4	Low	Slat		6 < 23	–	6 > (18 = 23)		–		–
4	High	Acla		6 < 18 < 23	(6 = 18) > 23	6 > 23	6 > (18 = 23)	6 > 23	(6 = 18) < 23	–
4	High	Slat		6 < 18 < 23	–	–		–		–
7	Low	Acla		–	–	–		–		–
7	Low	Slat		6 < 23	–	6 > 23		–		–
7	High	Acla		6 < 18 < 23	6 > 23	–	6 > (18 = 23)	6 > 23	(6 = 18) < 23	–
7	High	Slat		6 < 18 < 23	–	–		–	(6 = 18) < 23	–
10	Low	Acla		–	(6 = 23) < 18	18 > 6 > 23	6 < 23	6 < (18 = 23)		–
10	Low	Slat		6 < 23	–	–		–		–
10	High	Acla		6 < 18 < 23	(6 = 18) > 23	(6 = 23) < 18	6 > 18 > 23	6 > 23	6 < 18 < 23	–
10	High	Slat		6 < 18 < 23	–	(6 = 18) > 23		18 > 23		–

**Figure 2.**
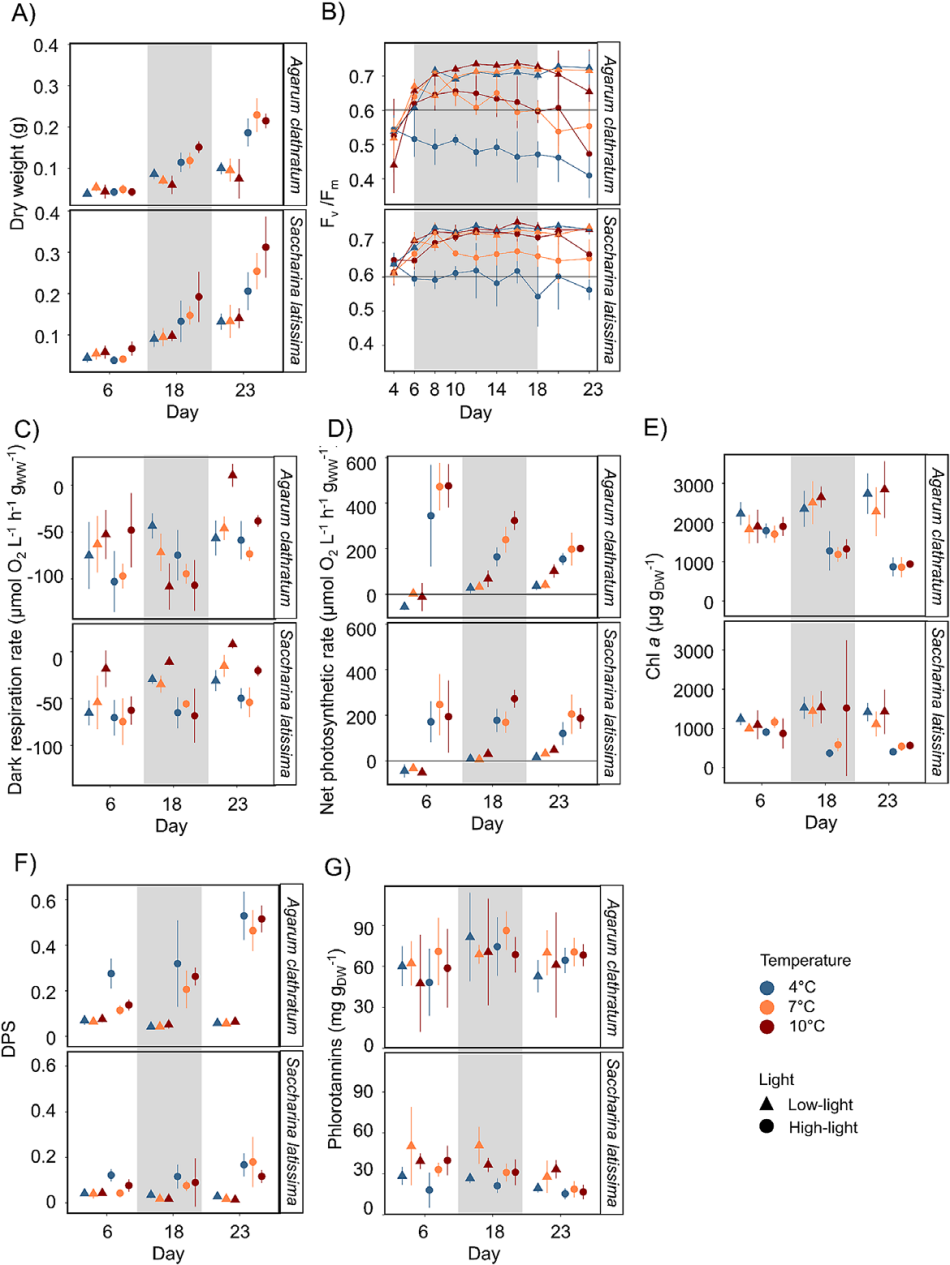
Physiological (A–D) and biochemical response (E–G) of *Agarum clathratum* and *Saccharina latissima* in the course of the experiment. Day 6: Wound healing and light acclimation. Day 18: heatwave (grey area). Day 23: recovery. Heatwave scenario: blue = 4°C; orange = 7°C; red = 10°C). Light conditions: triangle = low light, 3 μmol photon m^−2^ s^−1^; circle = high light, 120 μmol photons m^−2^ s^−1^ (*n* = 4). Statistical results of the pairwise comparisons are summarized in Table 2. (A) Dry weight (g). (B) Maximum quantum yield of photosystem II (F_v_/F_m_). (C) Dark respiration rate (μmol O_2_ L^−1^ h^−1^ g_WW_^−1^). Note that decreasing oxygen concentrations relate to increasing respiration rates. (D) Net photosynthetic rate (μmol O_2_ L^−1^ h^−1^ g_WW_^−1^). Horizontal black line: 0 μmol O_2_ L^−1^ h^−1^ g_WW_^−1^. (E) Chlorophyll *a* (μg g_DW_^−1^). (F) De-epoxidation state of xanthophyll cycle pigments (DPS). (G) Total phlorotannin concentration (mg g_DW_^−1^).

Growth (DW in g) is shown in Figure 2A. T, L, S, and D, as well as the interaction of T × L, significantly affected growth. Meristematic disks from both species showed no significant differences in DW at the beginning of the experiment. Both species gained more weight under high-light conditions compared to low-light conditions. Under high-light conditions, disks of both species became significantly heavier in the course of the experiment. Further, *Saccharina latissima* gained more weight at warm temperatures compared to 4°C. At 10°C, *S. latissima* was significantly heavier than *Agarum clathratum.*

Maximum quantum yield of photosystem II (F_v_/F_m_) (Figure 2B) was significantly affected by the fixed effects of T, L, S, and D, as well as the interaction between T × L and L × S × D. At the beginning of the experiment, F_v_/F_m_ of all treatments within one species were within the same range, or recovered until the start of the MHW. For both species, F_v_/F_m_ was lower under high light. This trend was intensified by cold temperatures and longer duration of the experiment. Generally, F_v_/F_m_ values were higher for *S. latissima* (0.543–0.744) than for *A. clathratum* (0.409–0.728).

The dark respiration rate (μmol O_2_ L^−1^ h^−1^ g_WW_^−1^; Figure 2C) was significantly influenced by T, L, S, and D as single fixed effects and T × S × D as interacting effect. For both species, dark respiration rates were lower under low-light compared to high-light conditions. At the peak of the MHW, the dark respiration rate of *A. clathratum* was highest, while after recovery it was lowest. In *S. latissima*, dark respiration rates were lowest under low-light conditions and 10°C throughout the experiment.

Net photosynthetic rates (μmol O_2_ L^−1^ h^−1^ g_WW_^−1^; Figure 2D) were significantly affected by T, L, and S, as well as L × S × D. Light treatments had the strongest effect on both species’ photosynthetic rates. Under high-light conditions, photosynthetic rates decreased during the experiment. Under low-light conditions, photosynthetic rates were negative before the MHW, increasing in the course of the experiment. Overall, the temperature effect on the photosynthetic rate was weak and only observed for *A. clathratum* at the beginning of the experiment (10°C > 4°C).

The chlorophyll *a* concentration (μg g_DW_^−1^; Figure 2E) was significantly affected by the fixed effects of L and S. In both species, chlorophyll *a* increased (trend) under low-light conditions, and decreased under high-light conditions in the course of the experiment (significant). Thereby, *A. clathratum* (1,788.4 ± 621 μg g_DW_^−1^) had significantly more chlorophyll *a* than *S. latissima* (1,041.1 ± 407 μg g_DW_^−1^).

The de-epoxidation state of xanthophyll cycle pigments (DPS; Figure 2F) was significantly affected by T, L, S, and D as well as the interactions of T × L and L × S × D. In both species, DPS was significantly lower under low-light conditions (0.043 ± 0.02) compared to high-light conditions (0.21 ± 0.15). Under low-light conditions, it did not change during the experiment in either species. Under high-light conditions, DPS was significantly higher in *A. clathratum* compared to *S. latissima.* The only temperature response was observed under high-light conditions, with 4°C being higher compared to warmer temperatures in *A. clathratum.*

The total phlorotannin content (mg g_DW_^−1^; Figure 2G) was affected by the single effects of T, S, and D. The mean phlorotannin content of *A. clathratum* of all treatments was 65.8 ± 10.2 mg g_DW_^−1^, thereby being about 150% higher than in *S. latissima* (30.5 ± 10.8 mg g_DW_^−1^), independently of the treatment.

**Figure 3.**
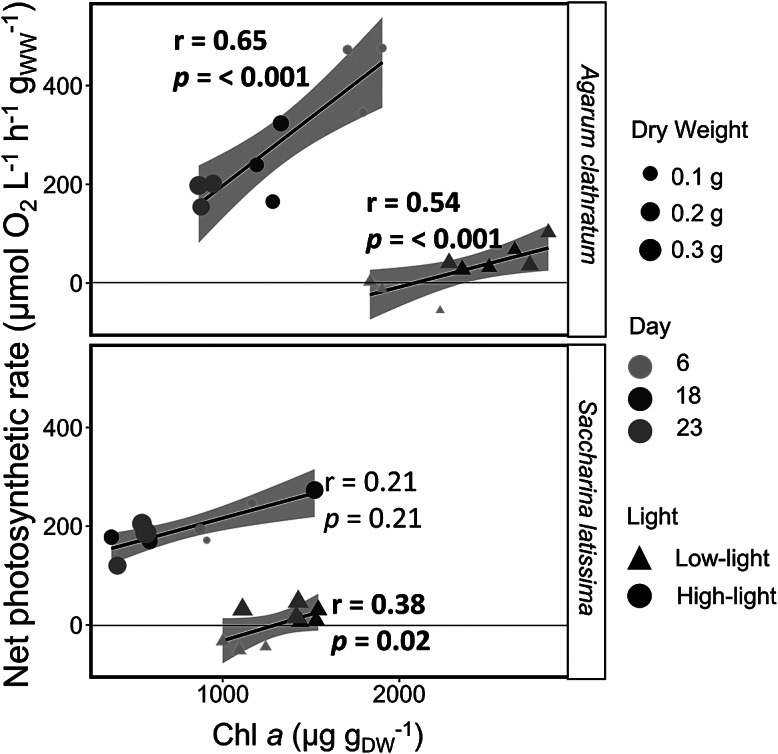
Linear dependency between the net photosynthetic rate (μmol O_2_ L^−1^ h^−1^ g_WW_^−1^) vs. chlorophyll *a* content (Chl*a*, μg g_DW_^−1^) of *Agarum clathratum* and *Saccharina latissima* after different temperature and light treatments. Size: Dry weight (g). Color: day of the experiment: 6 (orange), 18 (green), 23 (pink). Light conditions: low light (3 μmol photon m^−2^ s^−1^); high light (120 μmol photons m^−2^ s^−1^) (*n* = 4). Grey area: 95% confidence interval: *r*: Pearson correlation coefficient. Significant correlations (*p* < 0.05) are marked in bold.

In both species, the net photosynthetic rate correlated positively with the chlorophyll *a* content. This was significant in all cases, except for *S. latissima* under higher light conditions (*A. clathratum*: high light: ***p* = <0.001**, *t* = 5.01, *df* = 34; low light: ***p* = <0.001**, *t* = 3.75, *df* = 34; *S. latissima*: high light: *p* = 0.21, *t* = 1.27, *df* = 34; low light: ***p* = 0.02**, *t* = 2.36, *df* = 34). Under high-light conditions, the chlorophyll *a* concentration and net photosynthetic rate decreased in the course of the experiment. Under low-light conditions, chlorophyll *a* concentrations and net photosynthetic rates increased during the experiment (Figure 3). In all treatments, disks became heavier over time.

DPS of both species correlated negatively with F_v_/F_m_ in all treatments (*A. clathratum*: high light: ***p* = < 0.001**, *t* = −6.77, *df* = 34; low light: ***p* = 0.03**, *t* = −2.3, *df* = 34; *S. latissima*: high light: ***p* = 0.02**, *t* = −2.42, *df* = 34; low light: ***p* = 0.01**, *t* = −2.82, *df* = 34).

## Discussion

Due to ongoing climate change, the Arctic is one of the most affected and fastest changing regions in the world, with an increasing frequency of marine heatwaves (IPCC, 2023; Barkhordarian et al., 2024) and deteriorating light conditions in summer (Konik et al., 2021). In Arctic fjord systems, kelps act as foundation species and are challenged by changing conditions. Overall, kelps become more susceptible to additional stressors, when experiencing suboptimal temperature conditions (Wernberg et al., 2010). To be able to conserve these valuable ecosystems, it is important to understand how key species will respond to environmental changes (Lebrun et al., 2022), not only when single drivers change but also when multiple stressors interact. We designed a multifactorial experiment, comparing the reaction of two locally abundant kelp species (*Agarum clathratum*, *Saccharina latissima*) to summer heatwaves, when exposed to either low- or high-light conditions. The temperature and light treatments were based on measurements, reflecting MHWs in clear and meltwater dominated fjords (GEM, 2023; Niedzwiedz and Bischof, 2023b). In this setup, tested light amplitudes were the stronger driver (compared to temperature amplitudes), affecting kelp performance. We found *A. clathratum* to be low-light-adapted, confirming Hypothesis 1. Thereby, both species showed the potential to acclimate to varying light conditions by adjusting their pigment composition and concentration. Contradicting Hypothesis 2, the kelps showed no general positive response to increased temperatures, but only in combination with high-light conditions, mitigating physiological stress. Under low-light conditions, we detected no temperature effect, which we consider to be likely due to overall light limitation. Being exposed to high-light conditions, cold temperatures intensified physiological stress levels, contradicting Hypothesis 3. We consider this to be likely due to the photosynthetic electron transport chains being saturated earlier at cold temperatures.

### High-light stress is mitigated by warmer temperatures

In our experiment, both kelp species responded stronger to light variations than to the heatwave scenarios (Table 1). We based our treatment conditions on records by GEM (2023), classifying 4°C as summer in situ conditions (Supplement S2). Thereby, maximum quantum yield of photosystem II (F_v_/F_m_) was below 0.6 of all samples on day 4, indicating that field conditions caused emerging physiological and cellular stress levels in kelps (Dring et al., 1996; Murchie and Lawson, 2013). As optimum growth temperatures were reported between 10 and 15°C for both species (Bolton and Lüning, 1982; Egan et al., 1989; Simonson et al., 2015), this highlights the physiological limits at its cold distribution margins.

Under high-light conditions, F_v_/F_m_ further decreased in the course of the experiment, only recovering under low-light conditions (Figure 2B). Additionally, we measured significantly increasing DPS values in both species, when being exposed to high-light conditions (Figure 2F). The DPS of xanthophyll cycle pigments is an immediate reaction to protect the photosystem from the formation of oxidative stress by the interconversion of violaxanthin to antheraxanthin to zeaxanthin (Demmig-Adams and Adams, 1996). High DPS values have been described as response to high-light stress in kelps (Demmig-Adams and Adams, 1996) and correlating with decreasing F_v_/F_m_; this indicates increasing physiological stress levels under high-light conditions. As irradiances up to 186 μmol photons m^−2^ s^−1^ (max) at 15 m were measured in Nuup Kangerlua (Supplement S4), considering 120 μmol photons m^−2^ s^−1^ as “high” light seems contradictory. However, daily PAR cycles have to be considered. In our experiment, we mimicked polar day with 24 h constant light exposure to be able to draw general conclusions on Arctic ecosystems. At the sampling site on 15 m water depth, kelps were not exposed to PAR intensities >120 μmol photons m^−2^ s^−1^ for 24 h but rather 7.5 h (Supplement S4). Hence, the exposure of 120 μmol m^−2^ s^−1^ for 24 h, is causing high-light stress due to long photoperiod and the lack of a low-light recovery period. This emphasizes the necessity to not only consider mean PAR values but also variations in irradiance levels. Especially as variation in day length along the latitudinal gradient is one of the few factors that will not be affected by climate change, day length has to be considered to become a potential interacting stressor.

To mitigate high-light stress, both species acclimated by reducing their chlorophyll *a* concentration, which was significant for *A. clathratum.* This results in less electrons being transmitted into the electron transport chain, reducing the likelihood of reactive oxygen formation, and hence, oxidative stress (Kirk, 2011). The reduced chlorophyll *a* concentration correlated significantly with lower photosynthetic rates, despite which both species gained weight in the course of the experiment (Figure 3).

Thereby, the interaction of high-light conditions with high temperatures enhanced growth in *S. latissima.* As metabolic processes depend on a multitude of enzymatic reactions, they are characterized by the integration of the enzymatic properties, e.g., their temperature optimum for maximum capacity (Davison et al., 1991; Daniel et al., 2008). Highest growth rates for *S. latissima* were described between 10 and 15°C (Bolton and Lüning, 1982). Therefore, actual temperature conditions during sampling and control temperatures (4°C) are well below its optimum temperature. With increasing temperature, the enzymatic capacity is increasing (Pörtner et al., 2005), leading to higher potential growth rates. Accordingly, temperature-dependent increased respiratory losses have to be considered (Niedzwiedz and Bischof, 2023a). However, as *S. latissima* was not light-limited under high-light conditions, it accumulated carbon, resulting in enhanced weight gain. In *A. clathratum*, we detected no impact of temperature on growth at high-light conditions, even though the optimum growth temperature was also described between 10 and 15°C (Simonson et al., 2015) and disks were not light-limited (high photosynthetic rates). We attribute this to the overall high stress level experienced at high-light conditions and higher respiratory losses (Figure 2C), compared to *S. latissima.*

Under low-light conditions, the increase in weight was lower compared to high-light conditions. This can be explained by lower overall photosynthetic rates (Figures 2D and 3), i.e. less carbon accumulation (Kirk, 2011). Longer exposure to low-light intensities resulted in a low-light acclimation of both species: We measured an increase in chlorophyll *a* concentration that significantly correlated with higher photosynthetic rates to meet cellular energy demands, resulting in a net carbon gain. Overall, light limitation might be a reason why elevated temperatures did not boost growth under low-light conditions.

As both kelp species grew significantly over time in all experimental treatments (Figure 2A), we conclude that they were not starved of nutrients, even though low nutrient concentrations were measured for June water masses in Nuup Kangerlua (Juul-Pedersen et al., 2015).

### Herbivore deterrent in *A. clathratum*

Phlorotannins have been described as antioxidants that also have antimicrobial and antibacterial effects (Ford et al., 2019). Given the strong, correlating response in F_v_/F_m_ and DPS to high-light conditions, we expected higher phlorotannin concentrations under high-light compared to low-light conditions. However, we found no significant response of phlorotannins to light conditions. The only clear overall pattern we detected were mean phlorotannin concentrations of *A. clathratum* being more than twice as high as in *S. latissima.*

In addition to their antioxidant potential, phlorotannins are reported to respond to grazing pressure, acting as herbivore deterrent (Amsler et al., 2009). In the field, we observed high grazing pressure of sea urchins, e.g., *Strongylocentrotus droebachiensis*, on the kelp forest, forming sea urchin barrens. We were not able to quantify the effect of sea urchins on the kelp forests in the course of this study, but observed *S. latissima* to be completely absent on these sea urchin barrens, while *A. clathratum* was regularly found in small stands (Vonnahme, Niedzwiedz, pers. obs.). This interspecific difference in feeding preference was also described by Vadas (1977). A possible reason for that might be the high concentrations of phlorotannins in *A. clathratum.*

### Ecological implications – Plastic response

In our study, both kelp species showed stronger responses to the altered light conditions than to temperature changes. When discussing light conditions in Arctic fjords as consequence of climate change, different developments lead to opposing environmental conditions: Increased temperatures are leading to thinner and an early season breakup of sea ice, increasing the underwater irradiance (Nicolaus et al., 2012; Payne and Roesler, 2019). In combination with cold temperatures, our findings suggest that this seems to reduce the expansion of temperate kelp species to higher latitudes. Glacial melt (Milner et al., 2017), permafrost thaw (Bintanja, 2018) and increased precipitation rates (Bintanja and Andry, 2017) are leading to extensive sediment plumes in summer and darkening fjords (Gattuso et al., 2020; Konik et al., 2021). High terrestrial and glacial runoff rates increase the concentration of suspended particles in the water column, limiting the annual cumulative irradiance (Konik et al., 2021). We detected reduced weight gain of both kelp species, when cultivated under low-light conditions. Niedzwiedz and Bischof (2023a), Bartsch et al. (2016) and Düsedau et al. (n.d.) described the reduction of the kelp maximum distribution depth in meltwater plume dominated fjord systems, indicating reduced kelp primary production in darkening Arctic fjords. Schlegel et al. (2023) highlighted that the interannual in situ PAR variability is too large to project clear long-term developments of Arctic fjord light conditions, strongly depending on season, timescale, and boundary conditions. The high variability in in situ PAR in combination with the strong response of kelps to light variations indicates a high variability of temperate kelp presence along Arctic coastal areas. Thereby, we detected dynamic responses in chlorophyll *a* concentration responding to light conditions to maintain a positive net photosynthetic rate (low-light) or reduce oxidative stress (high-light). We conclude that both kelp species show a high grade of phenotypic plasticity, having the potential to acclimate to low-light conditions, and potentially compensating reduced production rates up to a certain degree. We suggest a long-term experiment specifically targeting the light-dependent production of different kelp species. Additionally, systematic and quantitative studies should be conducted, comparing and monitoring fjords in different stages of cryosphere loss, verifying the experimental results in nature.

In our setup, assessing the effect of summer MHWs in Arctic environments on cold-temperate kelp species, we detect no immediate negative interactions of warm temperature in combination with low-light conditions (Arctic coastal areas in the presence of a sediment plume). On the contrary, elevated temperatures in combination with high-light conditions seemed to have positive effects on the kelps physiological state (e.g., enhanced weight gain), possibly by increasing the enzymatic activities and thereby reducing photo-damaging. This can be explained by temperatures being closer to the species optimum growth temperature of 10–15°C (Bolton and Lüning, 1982; Egan et al., 1989; Simonson et al., 2015). However, these temperature ranges have been described for populations of lower latitudes and Bennett et al. (2019) review that intraspecific temperature susceptibility can change depending on geographic location. While we did not assess temperature reaction norms, our results do not contradict the reported optima, as high physiological stress levels were mitigated by warmer temperatures, being closer to 10–15°C. Thereby, we want to highlight that the positive effect of warmer temperatures during the marine heatwave under high-light stress on kelp physiology and biochemistry only holds true for temperate-adapted species on their cold-distribution edge. Highly detrimental effects of marine heatwaves on kelp forests and ecosystems have been described in other regions (e.g., Wernberg et al., 2013; Smale, 2019; Filbee-Dexter et al., 2020; Smith et al., 2023). As shown by Bass et al. (2023), the effect of sudden temperature increases in combination with varying light conditions on kelp species strongly depends on species and environmental conditions (temperature, light, season).

After conducting an Arctic summer experiment, we further want to highlight the necessity to explore effects of warming temperatures and MHWs during polar night. The studies by Gordillo et al. (2022) and Scheschonk et al. (2019) have described the detrimental effect of Arctic winter warming on kelps, given the monthlong period of darkness. Gagnon et al. (2005) reported *A. clathratum* growing during very low temperatures during spring and fall. Liesner et al. (2020) and Gauci et al. (2022) described reduced plasticity of *Laminaria digitata* when experiencing higher temperatures during development across generations and Martins et al. (2017) showed different thermal optima for different life stages.

Summarizing, we found light to induce stronger physiological and biochemical responses in both kelp species, with temperature either intensifying or mitigating the light-induced species-specific reactions. Thereby, *A. clathratum* was documented to be adapted to lower light intensities than *S. latissima,* enabling *A. clathratum* to maintain a positive net photosynthetic rate at lower light intensities. This enables *A. clathratum* to grow deeper in the water column than *S. latissima* (Vonnahme, Niedzwiedz, pers. obs. in Nuup Kangerlua), and further implying that it can grow earlier/later in season. However, we found *A. clathratum* to have the potential to acclimate to higher light intensities. Gagnon et al. (2005) reported *A. clathratum* to be very weak in the interspecific competition in shallow waters. Therefore, we hypothesize that *A. clathratum* might have been outcompeted to deeper water depths. Further, Gagnon et al. (2005) reported *A. clathratum* to establish in the presence of grazing pressure.

In an Arctic coastal area with warmer temperatures, less light due to higher sediment inputs and potentially higher grazing pressure, our findings suggest that *A. clathratum* will become more dominant at shallower depths, albeit at lower overall total biomass (assumption based on dry weight measurements). Ameralik fjord, a neighboring fjord to Nuup Kangerlua, represents this scenario (Meire et al., 2023). As expected, *A. clathratum* has indeed been found to acclimate to higher light conditions, occurring at depths up to the intertidal zone and becoming dominant over *S. latissima* (Vonnahme, pers. obs.). These conclusions add to the findings of Simonson et al. (2015), stating that at their warm distribution range (summer sea surface temperature: >18°C; Nova Scotia) *A. clathratum* might have a competitive advantage over *S. latissima* in the future. While the general ecosystem services of a kelp forest would prevail, changes in the kelp forests extent and species composition might have cascading effects on the entire ecosystem, entailing a change in biodiversity and biotic interactions of kelp associated species (Bégin et al., 2004; Smale et al., 2015) or a reduction of energy transfer to high trophic levels (Blain and Gagnon, 2014; Dethier et al., 2014).

## Supporting information

Niedzwiedz et al. supplementary material Niedzwiedz et al. supplementary material

## Data Availability

All data supporting the results of this study are openly available on the PANGAEA platform: https://doi.org/10.1594/PANGAEA.964643. Environmental data were provided by the marine monitoring program, MarinBasis-Nuuk, and climate monitoring program ClimateBasis Nuuk as part of the Greenland Ecosystem Monitoring program (http://www.g-e-m.dk; https://doi.org/10.17897/KMEK-TK21; https://doi.org/10.17897/8Z2W-D993), as well as Niedzwiedz and Bischof (2023b) (https://doi.org/10.1594/PANGAEA.951173).

## Supplementary material

The supplementary material for this article can be found at http://doi.org/10.1017/cft.2024.5.
