# Supplementary material for: Light-mediated temperature susceptibility of kelp species (Agarum clathratum, Saccharina latissima) in an Arctic summer heatwave scenario
Source: Camb Prism Coast Futur. 2024 Mar 13;2:e6. doi: 10.1017/cft.2024.5 (PMC12343920; doi:10.1017/cft.2024.5)
Supplement: Niedzwiedz et al. supplementary material [file S2754720524000052sup001.docx]

# **Supplementary material**

# **Title of Article:**

Light-mediated temperature susceptibility of kelp species (*Agarum clathratum*, *Saccharina latissima*) in an Arctic summer heatwave scenario

**Authors**:

Sarina Niedzwiedz^1*^, Tobias Reiner Vonnahme^2^, Thomas Juul-Pedersen^2^, Kai Bischof^1^, Nora Diehl^1*^

*corresponding authors: [sarina@uni-bremen.de](mailto:sarina@uni-bremen.de) / [ndiehl@uni-bremen.de](mailto:ndiehl@uni-bremen.de)

**Affiliation**:

^1^Marine Botany, Faculty of Biology and Chemistry & MARUM, University of Bremen, 28359 Bremen, Germany

^2^Greenland Climate Research Centre, Greenland Institute for Natural Resources (GINR), Nuuk, Greenland


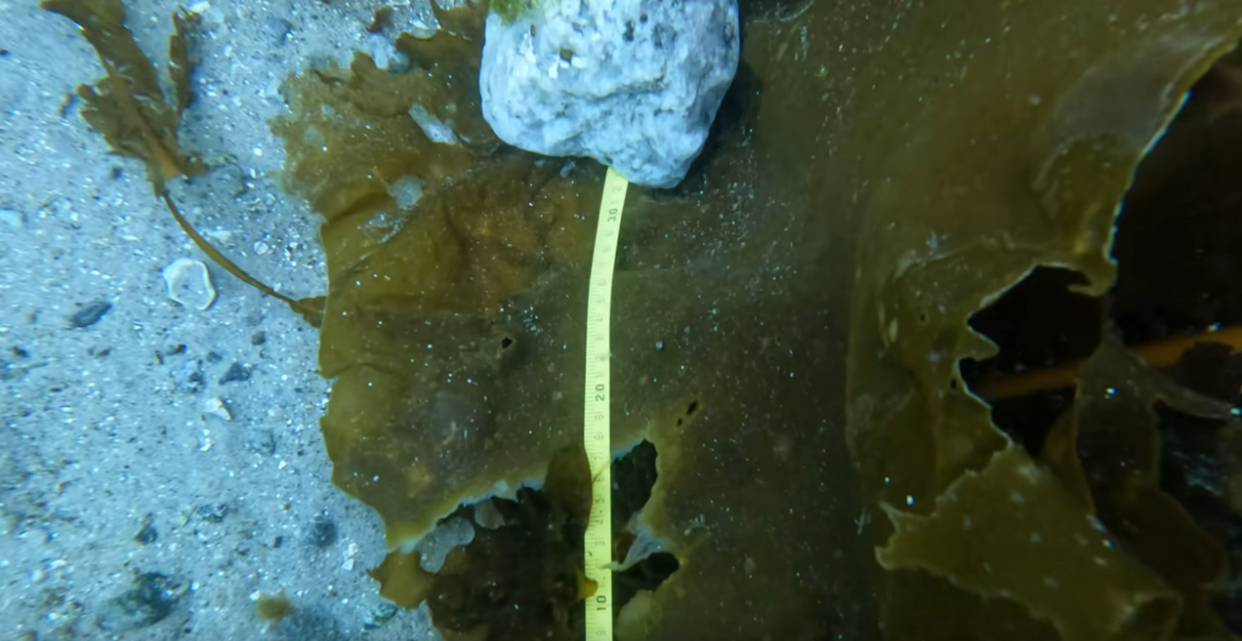


[https://youtu.be/TaZxT9NBcTM](https://www.google.com/url?q=https://youtu.be/TaZxT9NBcTM&sa=D&source=docs&ust=1708421196246047&usg=AOvVaw36mELY1tFZcJn8G4Ef9KNG)

**S1**: Video transect (by Tobias Reiner Vonnahme) through a kelp forest near Nuuk (01.05.2022) taken 0.5 m (+-0.5m) over the bottom or kelp canopy. The transect starts at about 12 m water depth and ends at the surface. Between ca.12 m and 10 m (2:08 min) the kelp species *Agarum clathratum*, *Saccharina latissima*, and *Alaria esculenta* dominate. Between ca. 5 m (2:35 min) and 2 m, *A. clathratum* is absent, while and first-year *A. esculenta* is most abundant. Laminaria sp. becomes increasingly abundant from ca 3-1m depth.


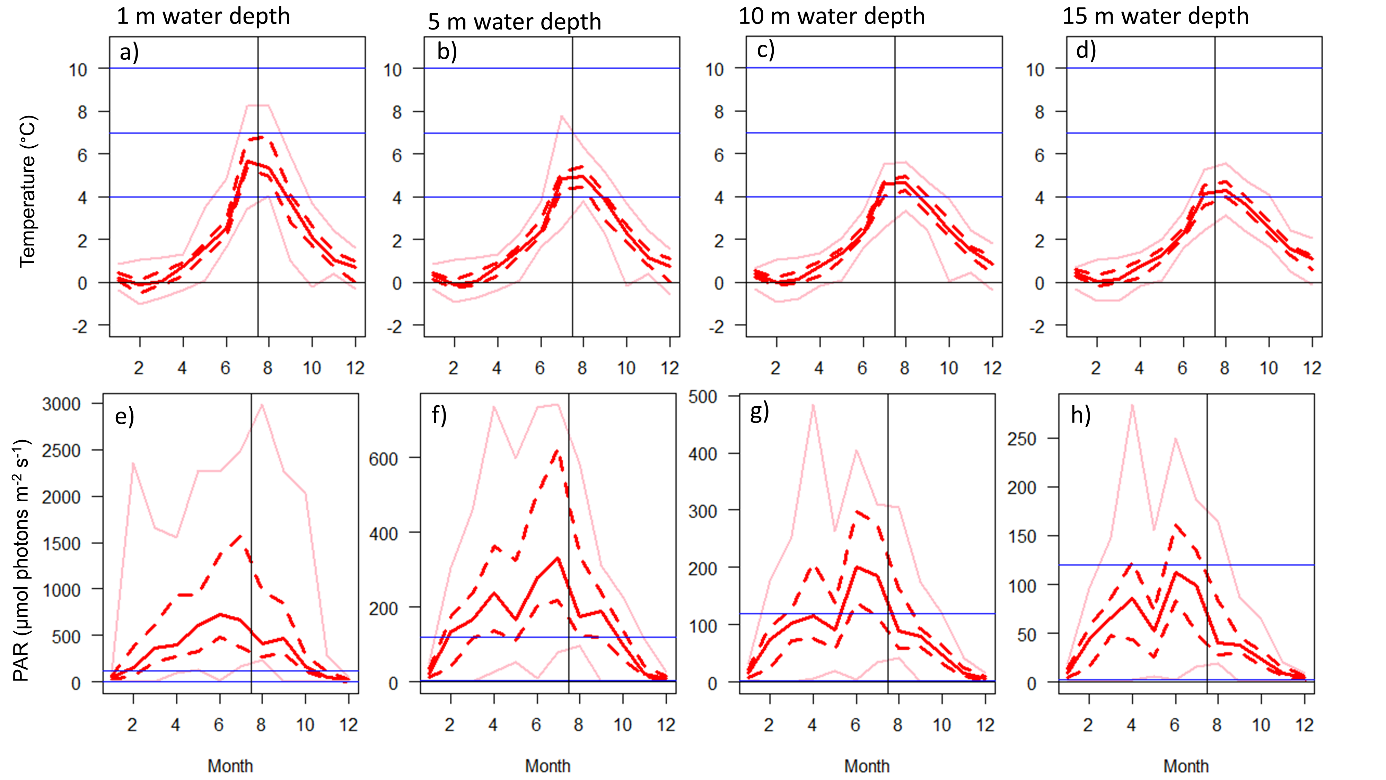


**S2**: Monthly temperature (a-d) and photosynthetically available radiation (PAR; µmol photons m^-2^ s^-1^) (e-h) from a marine monitoring station (GF3, G-E-M.dk/data; <https://doi.org/10.17897/KMEK-TK21>) showing median (solid red line), interquartile ranges (dotted lines), and the total range (light red) of the measurements at 1 m (a,e), 5 m (b,f), 10 m (c,g), and 15 m (d,h) depth between 2005 and 2021 at the nearby marine monitoring station (G-E-M.dk/data). Treatment light intensities and temperatures are shown as blue lines.


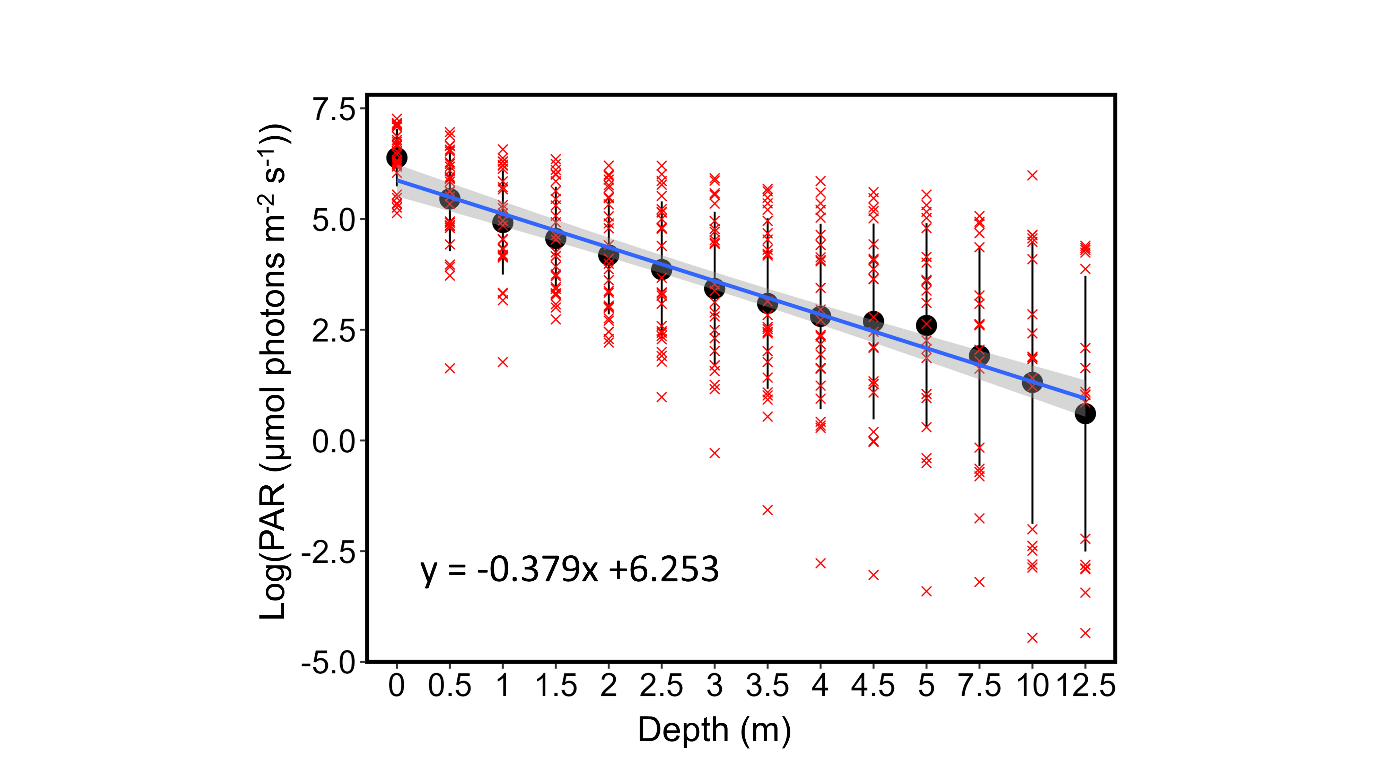


**S3**: Logarithm of photosynthetically available light (PAR) over water depth (m) in Kongsfjorden, Svalbard. Red crosses: single measurements. Black circles: Mean PAR±SD. Blue line: linear model of PAR intensities over depth ±95 % confidence interval. Data from Niedzwiedz & Bischof (2023b) <https://doi.org/10.1594/PANGAEA.951173>. The experiment's low-light conditions (3 µmol photons m^-2^ s^-1^) are at ~15 m water depth.


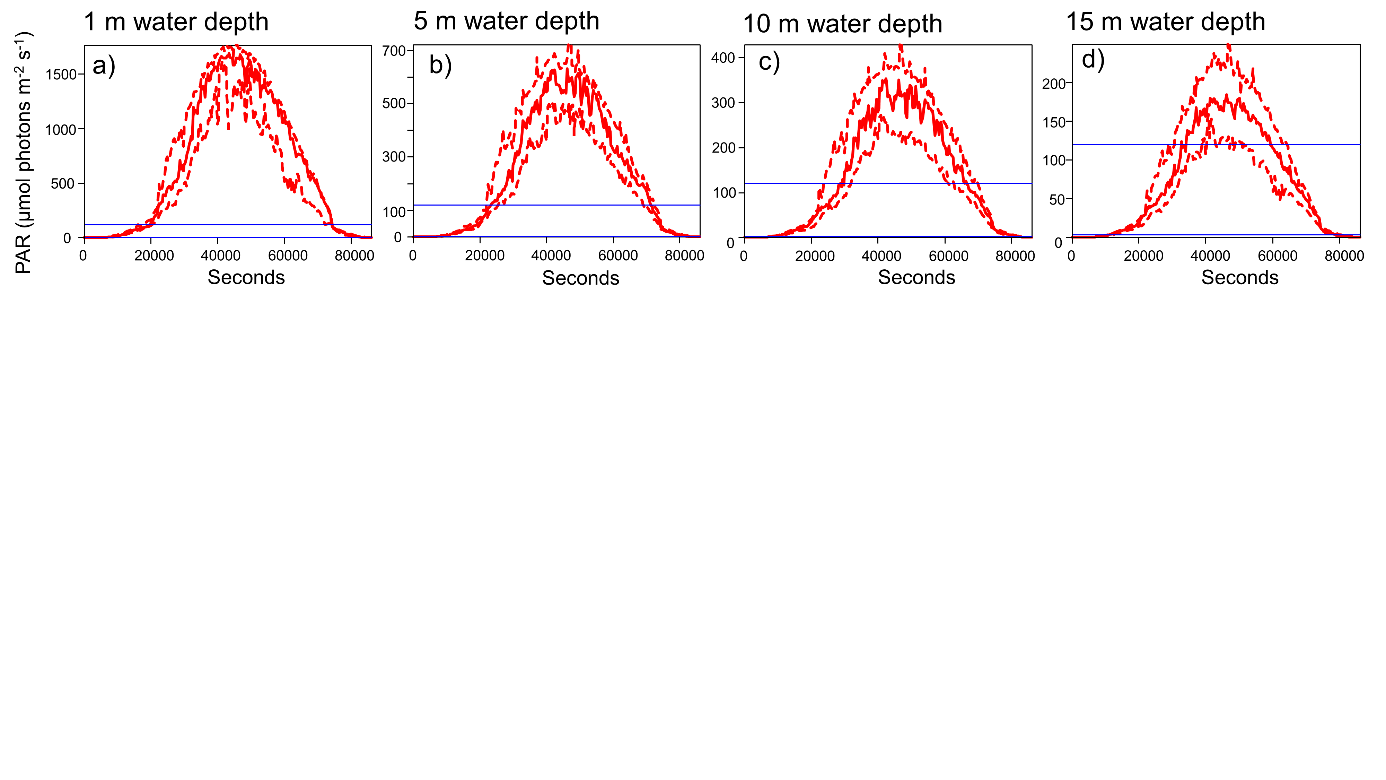


**S4**: Estimates of mean daily (sec) photosynthetically available radiation (PAR; µmol photons m^-2^ s^-1^) at 1 (a), 5 (b), 10 (c) and 15 (d) m depth in July since 2008 shown as median (solid red line) and interquartile ranges (dotted red line). Estimates are based on PAR measurements at 2 m over the bottom from ClimateBasis Nuuk (<https://doi.org/10.17897/8Z2W-D993>, Asiaq) and light attenuation based on PAR profiles measured at a nearby marine monitoring station (GF3, G-E-M.dk/data; <https://doi.org/10.17897/KMEK-TK21>). Treatment light intensities are shown as blue horizontal lines.
