# Supplementary material for: Light-mediated temperature susceptibility of kelp species (Agarum clathratum, Saccharina latissima) in an Arctic summer heatwave scenario
Source: Camb Prism Coast Futur. 2024 Mar 13;2:e6. doi: 10.1017/cft.2024.5 (PMC12343920; doi:10.1017/cft.2024.5)
Supplement: Peer review history

# Editor report: Recommendation: Light-mediated temperature susceptibility of kelp species (*Agarum clathratum*, *Saccharina latissima*) in an Arctic summer heatwave scenario — R0/PR2

**DOI:** 10.1017/cft.2024.5.pr2

**Affiliations:** Wildlife Conservation Society, United States

This study addressing how future climate scenarios are likely to impact two species of Artic kelps that are important foundation species for cold-temperature ecosystems is well suited for publication in Coastal Futures. The manuscript is well-written with a solid experimental design. I am particularly supportive suggestions by reviewer 2 to: (1) help improve readibility of the results (i.e., revise the results without linking them to day number); and (2) put the study in a broader global context of anticipated responses to marine heatwaves to better indicate potential differential responses in Arctic regions (e.g., comparison to trends observed from the literature from tropical and temperate ecosystems that show stronger negative impacts to macroalgae).

# Editor report: Decision: Light-mediated temperature susceptibility of kelp species (*Agarum clathratum*, *Saccharina latissima*) in an Arctic summer heatwave scenario — R0/PR3

**DOI:** 10.1017/cft.2024.5.pr3

**Affiliations:** Geography, Cambridge University, United Kingdom of Great Britain and Northern Ireland

No accompanying comment.

# Editor report: Recommendation: Light-mediated temperature susceptibility of kelp species (*Agarum clathratum*, *Saccharina latissima*) in an Arctic summer heatwave scenario — R1/PR5

**DOI:** 10.1017/cft.2024.5.pr5

**Affiliations:** Wildlife Conservation Society, United States

The authors have responded thoroughly to reviewers' queries. This will be a valuable contribution to Coastal Futures.

# Editor report: Decision: Light-mediated temperature susceptibility of kelp species (*Agarum clathratum*, *Saccharina latissima*) in an Arctic summer heatwave scenario — R1/PR6

**DOI:** 10.1017/cft.2024.5.pr6

**Affiliations:** Geography, Cambridge University, United Kingdom of Great Britain and Northern Ireland

No accompanying comment.
